# Enabling Early Obstructive Sleep Apnea Diagnosis With Machine Learning: Systematic Review

**DOI:** 10.2196/39452

**Published:** 2022-09-30

**Authors:** Daniela Ferreira-Santos, Pedro Amorim, Tiago Silva Martins, Matilde Monteiro-Soares, Pedro Pereira Rodrigues

**Affiliations:** 1 Department of Community Medicine, Information and Decision Sciences Faculty of Medicine, University of Porto Porto Portugal; 2 Center for Health Technology and Services Research Porto Portugal; 3 Sleep and Non-Invasive Ventilation Unit São João University Hospital Porto Portugal; 4 Portuguese Red Cross Health School Lisbon Lisbon Portugal

**Keywords:** machine learning, obstructive sleep apnea, systematic review, polysomnography

## Abstract

**Background:**

American Academy of Sleep Medicine guidelines suggest that clinical prediction algorithms can be used to screen patients with obstructive sleep apnea (OSA) without replacing polysomnography, the gold standard.

**Objective:**

We aimed to identify, gather, and analyze existing machine learning approaches that are being used for disease screening in adult patients with suspected OSA.

**Methods:**

We searched the MEDLINE, Scopus, and ISI Web of Knowledge databases to evaluate the validity of different machine learning techniques, with polysomnography as the gold standard outcome measure and used the Prediction Model Risk of Bias Assessment Tool (Kleijnen Systematic Reviews Ltd) to assess risk of bias and applicability of each included study.

**Results:**

Our search retrieved 5479 articles, of which 63 (1.15%) articles were included. We found 23 studies performing diagnostic model development alone, 26 with added internal validation, and 14 applying the clinical prediction algorithm to an independent sample (although not all reporting the most common discrimination metrics, sensitivity or specificity). Logistic regression was applied in 35 studies, linear regression in 16, support vector machine in 9, neural networks in 8, decision trees in 6, and Bayesian networks in 4. Random forest, discriminant analysis, classification and regression tree, and nomogram were each performed in 2 studies, whereas Pearson correlation, adaptive neuro-fuzzy inference system, artificial immune recognition system, genetic algorithm, supersparse linear integer models, and k-nearest neighbors algorithm were each performed in 1 study. The best area under the receiver operating curve was 0.98 (0.96-0.99) for age, waist circumference, Epworth Somnolence Scale score, and oxygen saturation as predictors in a logistic regression.

**Conclusions:**

Although high values were obtained, they still lacked external validation results in large cohorts and a standard OSA criteria definition.

**Trial Registration:**

PROSPERO CRD42021221339; https://www.crd.york.ac.uk/prospero/display_record.php?RecordID=221339

## Introduction

### Background

Obstructive sleep apnea (OSA) is a common sleep-related breathing disorder characterized by recurrent episodes of partial (hypopnea) or complete (apnea) upper airway obstruction, repeated throughout sleep. Its prevalence varies significantly according to how OSA is defined (methodology, criteria used such as apnea index, apnea-hypopnea index [AHI], or respiratory disturbance index and threshold definitions) and the population being studied [1]. The study by Benjafield et al [2] estimated that worldwide, 936 million adults aged 30 to 69 years have OSA. Despite this high prevalence, many cases remain undiagnosed and untreated, leading to a decrease in patients’ quality of life and an increased risk of adverse events, with a high impact on morbidity and mortality [3]. Polysomnography (PSG) is the gold standard test for diagnosing OSA [1]. However, performing PSG is costly, time-consuming, and labor-intensive. Most sleep laboratories face long waiting lists of patients, as PSG is neither a routine clinical practice nor an absolute suitable screening tool [4]. Given these limitations, it would be useful to develop a clinical prediction model that could reliably identify the patients most likely to benefit from PSG, that is, exclude OSA diagnosis when the probability is low, establish a priori probability before considering PSG, and prioritize patients in need of PSG according to the probability of a positive result. This idea was backed up by the American Academy of Sleep Medicine (AASM) in its latest guidelines [1]. Clinical prediction models should be easy to use and easy to calculate. The model must be based on the gold standard and required to be validated, and when used for screening, its purpose depends on whether the path leads to a rule-out or rule-in approach. In the first case, we should have a high-sensitivity model, omitting the need to perform PSG in healthy patients. By contrast, if we chose a rule-in approach, a high-specificity model is needed to select patients with a high probability of having OSA, suitable for undergoing PSG.

### Objective

Given these shortcomings, this systematic review aimed to identify, gather, and analyze existing machine learning approaches that are being used for disease screening in adult patients with suspected OSA.

## Methods

This systematic review was carried out according to a protocol registered with PROSPERO (International Prospective Register of Systematic Reviews; CRD42021221339).

### Search Strategy and Selection Criteria

We searched all evidence available in the MEDLINE database (PubMed) and in Scopus and ISI Web of Knowledge published until June 2020 in English, French, Spanish, or Portuguese. Specific queries were used (with a refresh in October 2021), and a manual search was also performed by using the references of the included studies and pertinent reviews on the topic. In addition, contact with specialists in the field was made to check whether all pertinent information was retrieved. Articles were selected by 3 reviewers independently (blinded to each other’s assessment) by applying the criteria to each title and abstract and then assessed fully. Divergent opinions were resolved through consensus. All processes were performed in Rayyan, a web application and mobile app for systematic reviews [5].

Studies including adult patients with suspected OSA (population) that assessed the accuracy of predictive models using known symptoms and signs of OSA (exposure and comparator) and had PSG as the gold standard (outcome) were eligible as per the selection criteria.

### Data Extraction

Once the articles were selected, data were extracted into a prespecified Excel spreadsheet and included (1) article information: title, author(s), publication date, country, and journal and (2) methods: study design, setting, study period, type of model, inclusion and exclusion criteria, participant selection, sample size, clinical factors analyzed, diagnostic test analyzed, and potential bias. For each type of model, specific data extraction was created and fulfilled, as demonstrated in the tables in further sections. We have ordered the identified studies by the obtained article results: first, the articles that only developed the algorithm; then the ones that internally validated the algorithm; and finally, the ones that externally validated the prediction algorithm. Within each subsection, we organized the published works by year of publication. Any missing information from the studies is reported in the Results section by “—” (not available), and the best obtained predictive model is marked in italic. Also, if the study applied different machine learning approaches, the clinical factors analyzed, and the discrimination measures are only described for the best obtained model.

### Risk of Bias

At 2 points in time, 1 reviewer assessed the risk of bias and applicability by applying the Prediction Model Risk of Bias Assessment Tool (PROBAST) to all the included studies. This is specific for studies developing, validating, or updating diagnostic prediction models. More details are available in the study by Moons et al [6]. An important aspect needs to be referred to, as this tool states that *“if a prediction model was developed without any external validation, and it was rated as low risk of bias for all domains, consider downgrading to high risk of bias. Such a model can only be considered as low risk of bias if the development was based on a very large data set and included some form of internal validation.”* This means that the included studies only performing model development will be marked as high risk of bias. For those with internal validation, the risk of bias will depend on the sample size based on the number of events per variable (≥20 ratio between events and variables in development studies and ≥100 participants with OSA for model validation studies). In addition, studies that randomly split a single data set into development and validation are considered as internal validation.

## Results

### Overview

**Figure 1:**
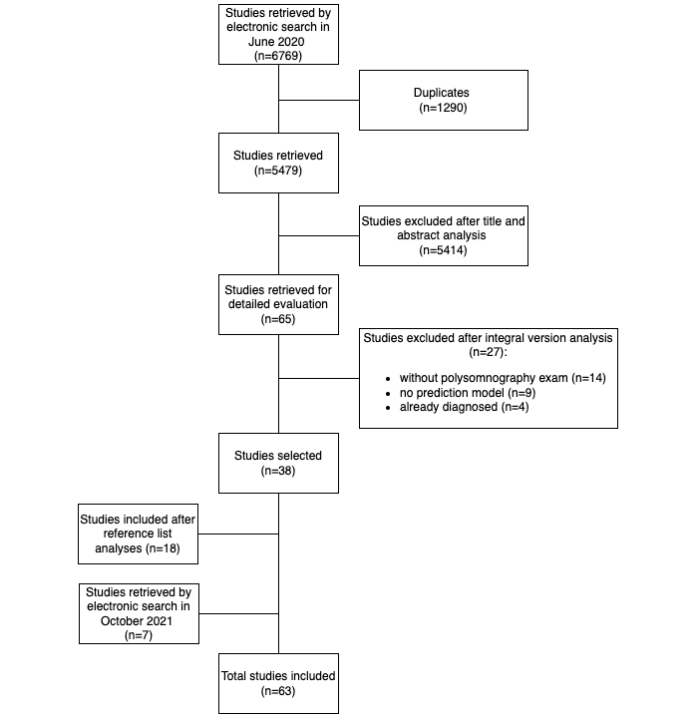
Flow diagram of the study selection process.

We retrieved 6769 articles, 1290 being duplicates. From the 5479 articles, we kept 63 studies that fulfilled the inclusion criteria, as shown in Figure 1.

The gold-standard examination—PSG—was performed in all the articles assessed, with one also adding the diagnostic part of the split-night exam [7]. The highest found age was 96 years [8], with 54% (34/63) of studies presenting patients with ages of >18 years. To be certain to include all OSA clinical prediction algorithms, we kept the studies that only reported a mean age and SD, with this value being >42, and SD varying between 6 and 16 years. In addition, 10% (6/63) of studies reported an age group <18 years (>14 and >15 years in 2/6, 33% studies and >16 and >17 in 4/6, 66% others, respectively). Regarding the suspicion of OSA, this description was shown in 65% (41/63) of studies, whereas 32% (20/63) introduced OSA suspicion and any other sleep disorder. In addition, we have a study with healthy patients and patients with suspected OSA [9] and another that does not specifically state this; instead, the authors write that patients already diagnosed with OSA were excluded from the study. The frequency of occurrence of the various clinical factors analyzed in more than 1 study is shown in Table 1.

There were disagreements between the reviewers in both phases, with an overall concordance rate of 78% in the title and abstract screening and 95% in the integral version.

**Table 1:** The frequency of occurrence of the various clinical factors analyzed that appears more than once in all the included studies (n=63).

Clinical factors analyzed	Frequency of occurrence, n (%)
BMI	37 (59)
Age	32 (51)
Sex	29 (46)
Neck circumference	25 (40)
Snoring	14 (22)
Epworth Somnolence Scale	10 (16)
Witnessed apneas	8 (13)
Waist circumference	8 (13)
Breathing cessation	7 (11)
Daytime sleepiness	7 (11)
Hypertension	7 (11)
Gasping	6 (10)
Oxygen saturation	6 (10)
Oxygen desaturation	6 (10)
Blood pressure	5 (8)
Smoking	5 (8)
Tonsil size grading	5 (8)
Modified Mallampati score	4 (6)
Alcohol consumption	3 (5)
Awakenings	3 (5)
Diabetes	3 (5)
Height	3 (5)
Nocturia	3 (5)
Restless sleep	3 (5)
Weight	3 (5)
Craniofacial abnormalities	2 (3)
Driving sleepy	2 (3)
Face width	2 (3)
Friedman tongue score	2 (3)
Snorting	2 (3)

### Prediction Models Development

New prediction models were developed in 23 studies, as presented and described in Table 2. The most common approach was regression techniques, with logistic (6/23, 26%), linear (6/23, 26%), logistic and linear (6/23, 26%), and logistic regression compared with decision trees and support vector machines (3/23, 13%). In addition, 4% (1/23) of articles produced a Pearson correlation and another (1/23, 4%) produced a decision tree. The oldest model was developed in 1991 and included sex, age, BMI, and snoring whereas in 2020 the predictive variables included besides these were height, weight, waist size, hip size, neck circumference (NC), modified Friedman score, daytime sleepiness, and Epworth Somnolence Scale score. Only 13% (3/23) studies described the study design and period, with 22% (5/23) being retrospective. Regarding OSA definition by PSG, 4% (1/23) study did not report the cutoff, while 17% (4/23) reported an AHI>10 and 17% (4/23) more reported an AHI≥15. The largest sample size was 953, and the smallest was 96 patients with suspected OSA. An overall prevalence of OSA between 31% and 87% was stated, with 9% (2/23) of studies presenting incorrect percentage values [10,11]. Regarding discrimination measures, although no validation was performed, the best area under the receiver operating characteristic curve (AUC), sensitivity, and specificity were 99%, 100%, and 95%, respectively. It should also be noted that 4% (1/23) has no mention of the best prediction model (not marked in italic in Table 2).

**Table 2:** Studies’ characteristics of prediction model development without internal or external validation with the best obtained model marked as italic in the respective model column.

Study	Study design; study period	Machine learning approach	Clinical factors analyzed	OSA^a^ definition	Sample size, n	OSA prevalence, n (%)	AUC^b^, % (95% CI)	Sensitivity, % (95% CI)	Specificity, % (95% CI)
Viner et al [12], 1991	Prospective; —^c^	Logistic regression	Sex, age, BMI, and snoring	AHI^d^>10	410	190 (46)	77 (73-82)	28 (—)	95 (—)
Keenan et al [13], 1993	—	Logistic regression	NC^e^, age, WA^f^, daytime sleepiness, driving sleepy, oxygen desaturation, and heart rate frequency	AHI>15	96	51 (53)	—	20 (—)	5 (—)
Hoffstein et al [14], 1993	—	Linear regression	Subjective impression	AHI>10	594	275 (46)	—	60 (—)	63 (—)
Flemons et al [15] 1994	—; February 1990 to September 1990	Logistic and *linear* regression	NC, hypertension, snoring, and gasping or choking	AHI>10	175	82 (46)	—	—	—
Vaidya et al [16], 1996	—; July 1993 to December 1994	*Logistic* and linear regression	Age, BMI, sex, and total number of symptoms	RDI^g^>10	309	226 (73)	—	96 (—)	23 (—)
Deegan et al [11], 1996	Prospective; —	Logistic and linear regression	Sex, age, snoring, WA, driving sleepy, alcohol consumption, BMI, number of dips ≥4%, lowest oxygen saturation, and NC	AHI≥15	250	135 (54)	—	—	—
Pradhan et al [17], 1996	Prospective; August 1994 to February 1995	Logistic regression	BMI, lowest oxygen saturation, and bodily pain score	RDI>10	150	85 (57)	—	100 (—)	31 (—)
Friedman et al [18], 1999	Prospective; —	Linear regression	Modified Mallampati class, tonsil size grading, and BMI	RDI>20	172	—	—	—	—
Dixon et al [19], 2003	—	*Logistic* and linear regression	BMI, WA, glycosylated hemoglobin, fasting plasma insulin, sex, and age	AHI≥30	99	36 (36)	91 (—)	89 (—)	81 (—)
Morris et al [10], 2008	Prospective; —	Pearson correlation	BMI and snoring severity score	RDI≥15	211	175 (83)	—	97 (—)	40 (—)
Martinez-Rivera et al [20], 2008	—	Logistic regression	Sex, waist-to-hip ratio, BMI, NC, and age	AHI>10	192	124 (65)	—	—	—
Herzog et al [21], 2009	Retrospective; —	Logistic and *linear* regression	Tonsil size grading, uvula size, dorsal movement during simulated snoring, collapse at tongue level, BMI, and ESS^h^ score	AHI>5	622	—	—	Female: 98 (—)	Female: 22 (—)
Yeh et al [22], 2010	Retrospective; April 2006 to December 2007	Linear regression	BMI, NC, and ESS score	AHI≥15	101	83 (82)	—	98 (—)	—
Hukins et al [23], 2010	Retrospective; January 2005 to July 2007	Linear regression	Mallampati class IV	AHI>30	953	297 (31)	—	40 (36-45)	67 (64-69)
Musman et al [24], 2011	—; December 2006 to March 2007	Logistic and *linear* regression	NC, WA, age, BMI, and allergic rhinitis	AHI>5	323	229 (71)	—	—	—
Sareli et al [25], 2011	—; November 2005 to January 2007	Logistic regression	Age, BMI, sex, and sleep apnea symptom score	AHI≥5	342	264 (77)	80 (—)	—	—
Tseng et al [26], 2012	—	Decision tree	Sex, age, preovernight systolic blood pressure, and postovernight systolic blood pressure	AHI≥15	540	394 (73)	—	—	—
Sahin et al [27], 2014	Retrospective; —	Linear regression	BMI, WC^i^, NC, oxygen saturation, and tonsil size grading	AHI>5 and symptoms	390	—	—	—	—
Ting et al [28], 2014	Prospective; —	Logistic regression and *decision trees*	Sex, age, and blood pressure	AHI≥15	540	394 (73)	99 (—)	98 (—)	93 (—)
Sutherland et al [29], 2016	—; 2011 to 2012	*Logistic* regression and classification and regression tree	Face width and cervicomental angle	AHI≥10	200	146 (73)	76 (68-83)	89 (—)	28 (—)
Lin et al [4], 2019	Retrospective; —	Linear regression	Sex, updated Friedman tongue position, tonsil size grading, and BMI	AHI≥5	325	283 (87)	80 (74-87)	84 (—)	58 (—)
Del Brutto et al [30], 2020	—	Logistic regression	Neck grasp	AHI≥5	167	114 (68)	62 (54-69)	83 (75-89)	40 (27-54)
Haberfeld et al [8], 2020	—	Logistic regression and *support vector machine*	Height, weight, WC, hip size, BMI, age, neck size, modified Friedman score, snoring, sex, daytime sleepiness, and ESS score	—	620	357 (58)	Male: 61 (—)	Male: 86 (—)	Male: 70 (—)

^a^OSA: obstructive sleep apnea.

^b^AUC: area under receiver operating characteristic curve.

^c^Not available.

^d^AHI: apnea-hypopnea index.

^e^NC: neck circumference.

^f^WA: witnessed apnea.

^g^RDI: respiratory disturbance index.

^h^ESS: Epworth somnolence scale.

^i^WC: waist circumference.

As stated in the Methods section, given that all these models only performed development with in-sample validation metrics, they were all considered at high risk of bias in the Analysis domain (Table 3). Concerning the Outcome domain, most studies were marked as high risk, as most of them did not have a prespecified or standard outcome definition. In addition, although some were marked as high risk and one as unclear, most included studies were at low risk of bias regarding the Predictors domain, showing that most of the studies did not include predictors after performing PSG. Most studies (15/23, 65%) were identified as unclear for the Participants domain, as almost all studies did not state study design or exclusion criteria. Assessing the applicability aspect of PROBAST, all studies (23/23, 100%) were at low risk of bias for the Participants domain (all studies included patients with suspected OSA), but several were at high risk of applicability for the Outcome domain (OSA definition is not in concordance with current OSA guidelines).

**Table 3:** Prediction Model Risk of Bias Assessment Tool (PROBAST) for prediction model development without internal or external validation.

Study	Risk of bias					Applicability				Overall	
	Participants	Predictors	Outcome	Analysis	Participants		Predictors	Outcome	Risk of bias		Applicability
Viner et al [12], 1991	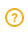 ^a^	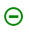 ^b^	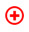 ^c^	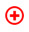	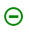		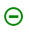	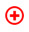	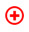		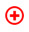
Keenan et al [13], 1993	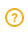	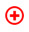	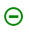	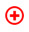	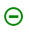		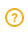	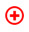	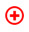		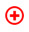
Hoffstein et al [14], 1993	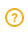	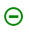	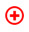	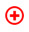	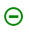		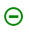	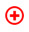	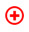		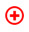
Flemons et al [15], 1994	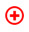	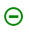	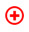	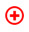	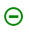		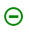	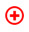	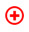		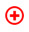
Vaidya et al [16], 1996	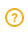	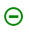	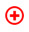	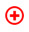	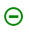		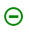	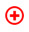	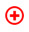		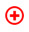
Deegan et al [11], 1996	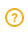	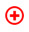	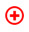	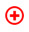	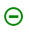		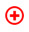	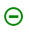	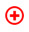		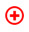
Pradhan et al [17], 1996	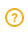	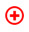	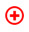	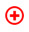	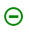		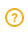	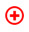	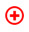		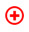
Friedman et al [18], 1999	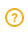	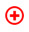	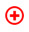	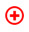	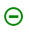		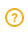	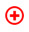	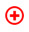		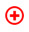
Dixon et al [19], 2003	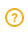	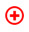	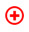	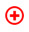	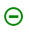		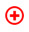	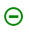	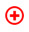		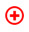
Morris et al [10], 2008	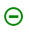	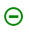	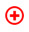	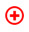	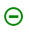		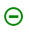	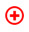	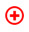		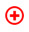
Martinez-Rivera et al [20], 2008	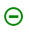	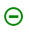	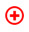	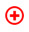	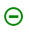		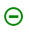	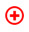	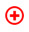		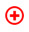
Herzog et al [21], 2009	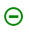	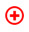	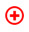	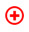	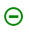		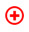	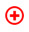	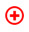		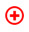
Yeh et al [22], 2010	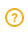	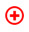	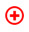	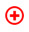	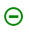		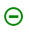	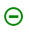	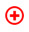		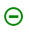
Hukins [23], 2010	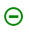	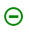	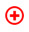	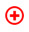	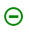		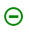	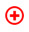	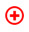		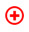
Musman et al [24], 2011	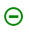	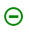	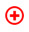	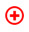	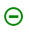		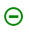	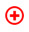	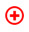		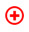
Sareli et al [25], 2011	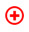	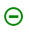	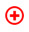	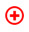	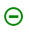		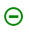	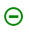	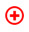		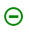
Tseng et al [26], 2012	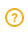	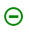	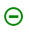	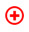	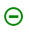		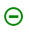	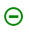	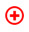		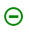
Sahin et al [27], 2014	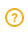	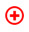	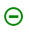	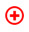	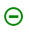		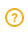	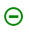	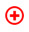		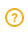
Ting et al [28], 2014	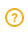	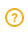	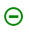	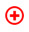	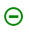		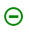	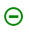	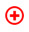		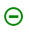
Sutherland et al [29], 2016	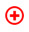	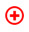	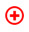	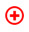	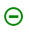		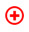	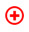	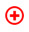		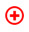
Lin et al [4], 2019	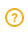	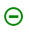	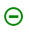	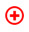	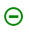		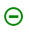	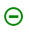	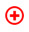		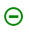
Del Brutto et al [30], 2020	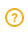	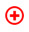	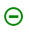	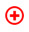	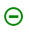		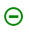	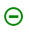	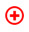		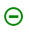
Haberfeld et al [8], 2020	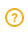	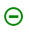	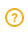	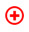	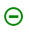		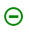	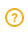	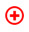		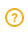

^a^Indicates an unclear risk of bias or concerns regarding applicability.

^b^Indicates a low risk of bias or concerns regarding applicability.

^c^Indicates a high risk of bias or concerns regarding applicability.

### Development of Prediction Models With Internal Validation

For purposes of internal validation, we considered studies that performed cross-validation (11/26, 42%), used bootstrapping techniques (4/26, 15%), or used split-data (14/26, 54%) as previously mentioned in the Methods section. The smallest sample size was 83 participants and the highest was 6399, with both presenting validation results for cross-validation. Regarding OSA prevalence, a study had no mention, and another demonstrated an incorrect value [31], whereas others had the lowest value at 30% and the highest at 90%. Different machine learning approaches were used, with the most common being support vector machines (4/26, 15%), followed by logistic regression (3/26, 12%). Moreover, 38% (10/26) of studies described the study type and period, with retrospective design being the most common.

In addition, Table 4 shows different OSA definitions, with 8% (2/26) of studies not reporting cutoff values and the most common definition being AHI≥5 (8/26, 31%), followed by AHI≥15 (5/26, 19%). It should be noted that although the studies indicated that some types of internal validation were performed, some did not present results (10/26, 38%).

Regarding discrimination measures for internal validation, the best AUC, sensitivity, and specificity were 97%, 99%, and 97%, respectively. The model with the best AUC included predictive variables collected from PSG, such as the arousal index, and was also the model with the best specificity. The best sensitivity value was obtained for the neural network model with 19 predictive variables included. A total of 4 studies reported a clinical cutoff, which allows potential clinical threshold importance, with 50% reported in 2 studies and 32% in the other two.

In contrast to Table 3, Table 5 demonstrated that although internal validation was performed, only 8% (2/26) of studies had a low risk of bias in the Analysis domain, the reason being not presenting the relevant calibration or discrimination measures, such as AUC, and using only *P* values to select predictors. Furthermore, in the Participants domain applicability, 8% (2/26) of studies were marked as having a high risk of applicability, as they did not select only patients with suspected OSA.

**Table 4:** Studies’ characteristics of prediction model development with internal validation. If the study applied different machine learning approaches, the clinical factors analyzed and the discrimination measures are only described for the best obtained model, marked as italic in the respective model column.

Study	Study design; study period		Machine learning approach		Clinical factors analyzed		OSA^a^ definition		Sample size, n		OSA prevalence, n (%)		AUC^b^, % (95% CI)			Sensitivity, % (95% CI)		Specificity, % (95% CI)
Kapuniai et al [9], 1988	—^c^		Discriminant analysis		Breathing cessation, adenoidectomy, BMI, and gasping		AI^d^>5		D_1_^e^=43; D_2_=53		13 (30)		—			61 (—)		67 (—)
Kirby et al [32], 1999	Retrospective; —		Neural network		Age, sex, frequent awakening, experienced choking, WA^f^, observed choking, daytime sleepiness, ESS^g^, hypertension, alcohol consumption, smoking, height, weight, BMI, blood pressure, tonsillar enlargement, soft-palate enlargement, crowding of the oral pharynx, and sum of the clinical scores for the binary categorical values		AHI^h^≥10		D_1_=255; D_2_=150		281 (69)		94 (—)			99 (97-100)		80 (70-90)
Lam et al [33], 2005	Prospective; January 1999 to December 1999		Discriminant analysis		Mallampati score, thyromental angle, NC^i^, BMI, age, and thyromental distance		AHI≥5		D_1_=120; D_2_=119^j^		201 (84)		71 (—)^k^			—		—
Julià-Serdà et al [34], 2006	—		Logistic regression		NC, sex, desaturation, ESS score, and distance between the gonion and the gnathion		AHI≥10		D_1_=150; D_2_=57		115 (56)		97 (95-99)^k^			94 (—)		83 (—)
Polat et al [35], 2008	Prospective; —		*Decision tree*, neural network, 21 adaptive neuro-fuzzy inference system, and artificial immune recognition system		Arousals index, AHI, minimum oxygen saturation value in stage REM^l^, and percentage of sleep time in stage of oxygen saturations intervals bigger than 89%		AHI>5		D_1_=41; D_2_=42^j^		58 (70)		97 (—)			92 (—)		97 (—)
Chen et al [31], 2008	—; January 2004 to December 2005		Support vector machine		Oxygen desaturation index		AHI≥5		566^j^		491 (87)		—			43 (—)		94 (—)
Lee et al [36], 2009	Prospective; —		*Logistic regression* and classification and regression tree		Face width, eye width, mandibular length, WA, and modified Mallampati class		AHI≥10		180^j^		114 (63)		87 (—)^k^			85 (—)^k^		70 (—)^k^
Rofail et al [37], 2010	—; July 2006 to November 2007		Logistic regression		Index 1 (snoring, breathing cessation, snorting, gasping), and nasal flow RDI^m^		AHI≥5		D_1_=96; D_2_=97		139 (72)		89 (81-97)			85 (—)		92 (—)
Chen et al [38], 2011	Retrospective; —		Logistic regression		Desaturation 3%		RDI≥30		D_j_=355; D_2_=100^j^		307 (86)		95 (—)^k^			90 (—)		90 (—)
Bucca et al [39], 2011	Prospective; January 2004 to December 2005		Linear regression		Age, NC, BMI, FEF50/FIF50^n^, COH_B_%^o^, smoking, F_eNO_^p^, and interaction smoking and F_eNO_		AHI≥30		201^q^		120 (60)		—			—		—
Bouloukaki et al [40], 2011	Prospective; October 2000 to December 2006		Linear regression		NC, sleepiness severity, BMI, and sex		AHI≥15		D_1_=538; D_2_=2152		2130 (79)		78 (61-80)^k^			70 (—)^k^		73 (—)^k^
Sun et al [41], 2011	—; February 2009 to June 2009		Logistic regression and *genetic algorithm*		Demographic data, ESS, systemic diseases, snoring, and comorbidities		AHI≥15		D_1_=67; D_2_=43		53 (48)		—			82 (—)		95 (—)
Laporta et al [42], 2012	Prospective; October 2010 to September 2011		Neural network		Age, weight, sex, height, NC, hypertension, daytime sleepiness, difficulty falling asleep, snoring, breathing cessation, restless sleep, and gasping		AHI≥5		91^q^		68 (75)		93 (85-97)^k^			99 (92-100)^k^		87 (66-97)^k^
Hang et al [43], 2013	Retrospective; January 2005 to December 2006		Support vector machine		Oxygen desaturation index, ESS, or BMI		AHI≥15		D_1_=188; D_2_=188; D_3_=189		—		—			88 (85-90)^k^		90 (87-94)^k^
Hang et al [44], 2015	—; January 2004 to December 2005		Support vector machine		Oxygen desaturation index		AHI>30		1156^j^		285 (46)		D_1_: 96 (—)^k^; D_2_: 95 (—)^k^			D_1_: 87 (—); D_2_: 91 (—)^k^		D_1_: 93 (—); D_2_: 90 (—)^k^
Ustun et al [7], 2016	—; January 2009 to June 2013		Logistic regression, *supersparse linear integer models*, decision tree, and support vector machines		Age, sex, BMI, diabetes, hypertension, and smoking		AHI>5		1922^j^		1478 (77)		79 (—)			64 (—)		23 (—)
Bozkurt et al [45], 2017	Retrospective; January 2014 to August 2015		Logistic regression, *Bayesian network*, decision tree, random forest, and neural network		Sex, age, BMI, NC, and smoking		AHI≥5		338^j^		304 (90)		73 (—)			86 (—)		85 (—)
Ferreira-Santos [46], 2017	Retrospective; January 2015 to May 2015		Bayesian network		Sex, NC, CFA^r^, WA, nocturia, alcohol consumption, ESS, concentration decrease, atrial fibrillation, stroke, myocardial infarction, driver, and daytime sleepiness		AHI≥5		194^j^		128 (66)		76 (73-78)			81 (79-83)		48 (44-51)
Liu et al [47], 2017	—; October 2005 to April 2014 and October 2013 to September 2014		Support vector machine		WC^s^, NC, BMI, and age		AHI≥15		6399^j^		3866 (60)		Female: 90 (87-94)			Female: 83 (75-91)		Female: 86 (82-90)
Manoochehri et al [48], 2018	—; 2012 to 2016		Logistic regression and *decision tree*		WC, snoring, sex, sleep apnea, ESS score, and NC		—		D_1_=239; D_2_=99		208 (62)		—			67 (—)		81 (—)
Manoochehri et al [49], 2018	—; 2012 to 2015		Logistic regression and *support vector machine*		Age, sex, BMI, NC, WC, tea consumption, smoking, hypertension, chronic headache, heart disease, respiratory disease, neurological disease, and diabetes		—		D_1_=176; D_2_=74		154 (62)		—			71 (—)^k^		85 (—)^k^
Xu et al [50], 2019	—; 2007 to 2016		Nomogram		Age, sex, glucose, apolipoprotein B, insulin, BMI, NC, and WC		AHI>5		4162^q^		3387 (81)		84 (83-86)			77 (76-79)^k^		76 (72-80)^k^
Ferreira-Santos et al [51], 2019	Retrospective; January 2015 to May 2015		Bayesian network		Sex, WA, age, nocturia, CFA, and NC		AHI≥5		194^j^		128 (66)		64 (61-66)			90 (88-92)		24 (20-27)
Keshavarz et al [52], 2020	Retrospective; February 2013 to December 2017		Logistic regression, Bayesian network, *neural network*, k-nearest neighbors, support vector machine, and random forest		Snoring, nocturia, awakening owing to the sound of snoring, snoring, back pain, restless sleep, BMI, and WA		AHI>15		231^j^		152 (66)		75 (—)			86 (—)		53 (—)
Chen et al [53], 2021	Retrospective; September 2015 to January 2020		Nomogram		Age, sex, snoring, type 2 diabetes mellitus, NC, and BMI		AHI≥5		D_1_=338; D_2_=144^q^		342 (71)		83 (76-90)			69 (63-75)^k^		87 (79-93)^k^
Hsu et al [54], 2021	—; December 2011 to August 2018		*Logistic regression*, support vector machine, and neural network		Sex, age, and BMI		AHI≥15		D_1_=2446; D_2_=1049		2539 (73)		82 (—)			73 (—)^k^		77 (—)^k^

^a^OSA: obstructive sleep apnea.

^b^AUC: area under receiver operating characteristic curve.

^c^Not available.

^d^AI: apnea index.

^e^D_1_, D_2_, and D_3_: data set.

^f^WA: witnessed apnea.

^g^ESS: Epworth somnolence scale.

^h^AHI: apnea-hypopnea index.

^i^NC: neck circumference.

^j^cross-validation.

^k^Internal derivation results.

^l^REM: rapid eye movement.

^m^RDI: respiratory disturbance index.

^n^FEF50/FIF50: forced midexpiratory/midinspiratory airflow ratio.

^o^COHB%: carboxyhemoglobin percent saturation.

^p^Fe_NO_: exhaled nitric oxide.

^q^Bootstrapping.

^r^CFA: craniofacial and upper airway.

^s^WC: waist circumference.

**Table 5:** Prediction Model Risk of Bias Assessment Tool (PROBAST) for prediction model development with internal validation.

Study	Risk of bias					Applicability				Overall	
	Participants	Predictors	Outcome	Analysis	Participants		Predictors	Outcome	Risk of bias		Applicability
Kapuniai et al [9], 1988	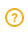 ^a^	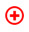 ^b^	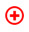	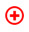	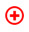		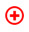	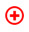	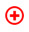		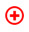
Kirby et al [32], 1999	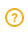	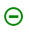 ^c^	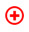	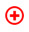	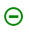		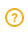	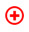	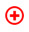		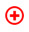
Lam et al [33], 2005	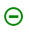	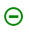	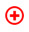	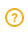	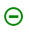		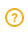	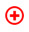	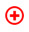		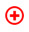
Julià-Serdà et al [34], 2006	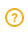	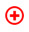	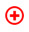	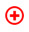	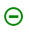		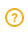	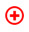	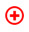		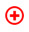
Polat et al [35], 2008	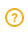	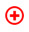	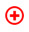	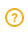	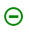		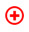	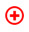	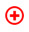		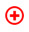
Chen et al [31], 2008	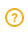	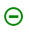	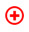	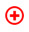	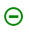		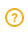	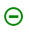	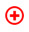		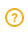
Lee et al [36], 2009	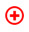	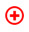	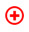	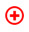	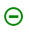		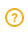	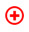	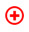		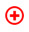
Rofail et al [37], 2010	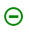	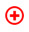	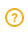	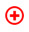	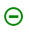		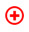	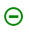	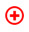		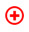
Chen et al [38], 2010	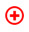	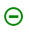	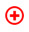	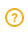	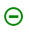		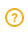	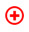	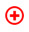		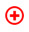
Bucca et al [39], 2010	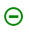	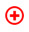	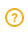	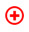	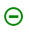		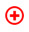	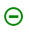	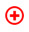		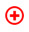
Bouloukaki et al [40], 2011	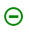	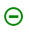	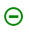	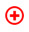	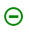		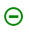	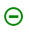	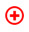		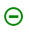
Sun et al [41], 2011	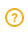	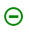	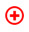	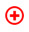	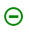		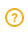	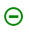	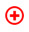		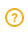
Laporta et al [42], 2012	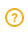	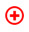	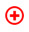	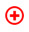	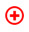		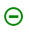	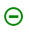	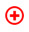		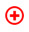
Hang et al [43], 2015	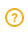	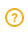	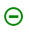	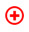	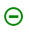		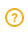	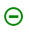	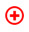		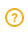
Hang et al [44], 2015	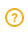	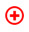	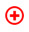	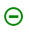	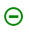		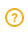	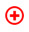	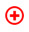		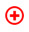
Ustun et al [7], 2016	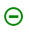	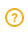	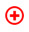	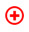	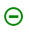		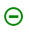	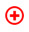	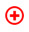		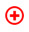
Bozkurt et al [45], 2017	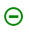	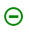	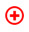	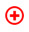	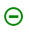		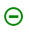	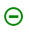	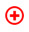		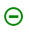
Ferreira-Santos et al [46], 2017	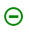	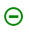	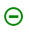	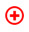	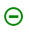		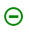	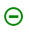	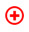		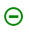
Liu et al [47], 2017	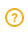	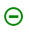	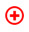	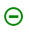	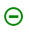		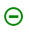	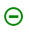	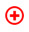		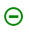
Manoochehri et al [48], 2018	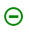	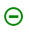	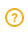	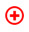	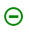		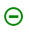	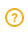	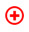		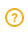
Manoochehri et al [49], 2018	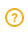	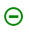	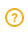	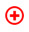	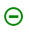		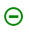	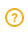	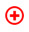		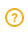
Xu et al [50], 2019	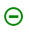	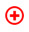	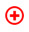	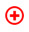	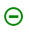		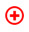	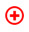	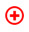		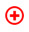
Ferreira-Santos et al [51], 2019	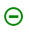	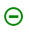	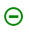	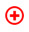	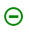		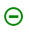	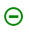	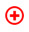		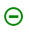
Keshavarz et al [52], 2020	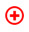	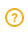	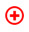	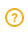	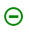		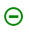	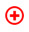	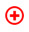		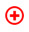
Chen et al [53], 2021	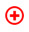	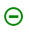	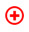	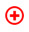	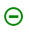		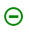	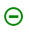	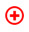		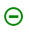
Hsu et al [54], 2021	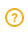	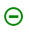	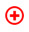	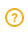	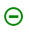		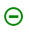	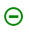	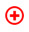		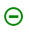

^a^Indicates an unclear risk of bias or concerns regarding applicability.

^b^Indicates a high risk of bias or concerns regarding applicability.

^c^Indicates a low risk of bias or concerns regarding applicability.

### Development of Prediction Models With External Validation

A total of 12 studies performed external validation, as described in Table 6, with 9 (75%) of them choosing logistic regression for the machine learning approach. The other 25% (3/12) elected linear regression, neural networks, or both. Regarding the study design, 3 (25%) studies elected a prospective design for testing and validation and 8% (1/12) of studies for only validation. Similar to the studies that only performed internal validation, the lowest OSA prevalence was 30%, and the highest was 93%, with a sample size varying between 169 and 3432 participants with suspected OSA. The best discriminatory model was logistic regression; it included age, waist circumference, ESS, and minimum oxygen saturation, with an AUC of 0.98 (0.96-0.99), for an OSA definition of AHI≥5. The higher reached sensitivity (100%) was also for a logistic regression but for a cutoff of AHI≥15, including specific respiratory conductance and daytime arterial oxygen saturation. The study also presented a clinical cutoff of 50%. Concerning specificity, the value of 94% was the highest for an AI>10, with self-reporting apneas, NC index, age, and tendency to fall asleep unintentionally as predictive variables.

As shown in Table 7, which aggregates information from the test and validation data sets, most studies were marked as unclear risk of bias in the Participants domain, as the studies referred to the study design for the test population but not for the validation data set. In addition, only 17% (2/12) of studies had a high risk of bias for the Predictors domain, given that the predictors could take time to be assessed or collected. Regarding the Analysis domain, half (6/12, 50%) of the studies were marked as having a low risk of bias, with 33% (4/12) of studies not presenting adequate performance metrics. The applicability in the Predictors domain is unclear in 8% (1/12) of studies, as we cannot assess whether the predictors are available in primary health care.

**Table 6:** Studies’ characteristics of prediction model development with external validation. If the study applied different machine learning approaches, the clinical factors analyzed and the discrimination measures are only described for the best obtained model, marked as italic in the respective model column.

Study	Study design; study period	Machine learning approach	Clinical factors analyzed	OSA^a^ definition	Sample size, n	OSA prevalence, n (%)	AUC^b^, % (95% CI)	Sensitivity, % (95% CI)	Specificity, % (95% CI)
Crocker et al [55], 1990	—^c^; October 1986 to May 1988	Logistic regression	Age, breathing cessation, BMI, and hypertension	AHI^d^>15	T^e^=100; V^f^=105	62 (30)	—	92 (—)	51 (—)
Pillar et al [56], 1992	—	Logistic regression	WA^g^, NC^h^ index, age, daytime and sleepiness	AI^i^>10 and symptoms	*t*=86; V_1_=50; V_2_=105	—	—	V_1_=88 (—); V_2_=32 (—)	V_1_=25 (—); V_2_=94 (—)
Maislin et al [57], 1995	—	Logistic regression	BMI, age, sex, index 1 (snoring, breathing cessation, snorting, and gasping), and BMI index 1	RDI^j^≥10	*t*=658; V=193	760 (89)	79 (—)^k^	—	—
Kushida et al [58], 1997	Prospective; 6 months (V)	Linear regression	Palatal height, maxillary intermolar distance, mandibular intermolar distance, overjet, BMI, and NC	RDI≥5	*t*=30; V=300^l, m^	254 (85)	100 (—)^k^	98 (95-99)^k^	100 (92-100)^k^
El-Solh et al [59], 1999	Retrospective (T) and prospective (V); November 1995 to December 1996	*Neural network* and linear regression	Breathing cessation, restless sleep, decreased libido, disturbs bed partner, daytime sleepiness, restless legs, BMI, NC, age, gasping, snoring, and blood pressure	AHI>10	*t*=189^l^; V=80	182 (68)	96 (93-96)	95 (90-98)^k^	65 (50-78)^k^
Zerah-Lancner et al [60], 2000	Retrospective (T) and prospective (V); —	Logistic regression	Specific respiratory conductance and daytime arterial oxygen saturation	AHI≥15	*t*=168; V=101	147 (55)	—	100 (—)	84 (—)
Rodsutti et al [61], 2004	Prospective; February 2001 to April 2003	Logistic regression	Age, sex, BMI, and breathing cessation	AHI≥5	*t*=837; V=243	569 (53)	79 (—)	—	—
Khoo et al [62], 2011	—; December 2005 to December 2007 and March 2008 to June 2008	Logistic regression	Sex, age, NC, and frequent awakening with unrefreshing sleep	AHI≥20	*t*=117; V=52	77 (66)	69 (—)^k^	78 (—)	45 (—)
Zou et al [63], 2013	Retrospective; January 2007 to July 2011	Logistic regression	Age, WC^n^, ESS^o^, and minimum oxygen saturation	AHI≥5	*t*=2052; V=784	2451 (87)	98 (96-99)	94 (92-96)	86 (79-91)
Karamanli et al [64], 2016	Retrospective; —	Neural network	Sex, age, BMI, and snoring	AHI≥10	*t*=201; V=15	140 (70)	—	—	—
Tawaranurak et al [65], 2020	Prospective; June 2018 to June 2020	Logistic regression	Sex, choking or apnea, blood pressure, NC, WC, and BMI	AHI≥15	*t*=892; V=374	826 (93)	75 (—)^k^	93 (89-96)	26 (18-35)
Park et al [66], 2021	—; January 2011 to December 2018	Logistic regression	Age, sex, BMI, hypertension, Berlin questionnaire score, and tonsil grade	AHI≥5	*t*=2516; V=916	—	84 (—)	78 (—)	76 (—)

^a^OSA: obstructive sleep apnea.

^b^AUC: area under receiver operating characteristic curve.

^c^Not available.

^d^AHI: apnea-hypopnea index.

^e^T: test data set.

^f^V: validation data set.

^g^WA: witnessed apnea.

^h^NC: neck circumference.

^i^AI: apnea index.

^j^RDI: respiratory disturbance index.

^k^Internal derivation results.

^l^Cross-validation.

^m^Bootstrapping.

^n^WC: waist circumference.

^o^ESS: Epworth Somnolence Scale.

**Table 7:** Prediction Model Risk of Bias Assessment Tool (PROBAST) for prediction model development with external validation.

Study	Risk of bias					Applicability				Overall	
	Participants	Predictors	Outcome	Analysis	Participants		Predictors	Outcome	Risk of bias		Applicability
Crocker et al [55], 1990	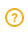 ^a^	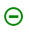 ^b^	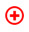 ^c^	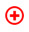	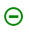		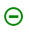	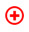	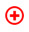		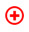
Pillar et al [56], 1994	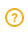	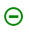	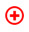	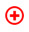	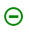		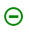	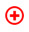	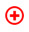		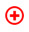
Maislin et al [57], 1995	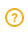	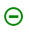	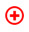	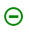	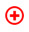		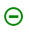	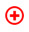	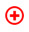		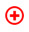
Kushida et al [58], 1997	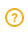	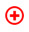	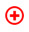	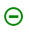	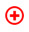		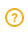	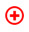	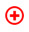		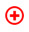
El-Solh et al [59], 1999	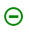	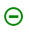	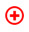	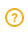	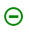		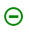	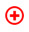	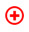		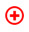
Zerah-Lancner et al [60] 2000	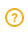	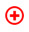	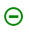	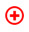	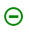		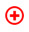	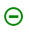	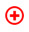		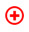
Rodsutti et al [61], 2003	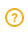	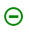	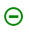	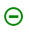	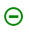		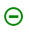	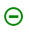	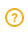		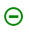
Khoo et al [62], 2011	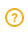	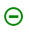	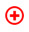	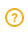	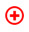		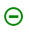	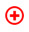	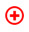		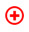
Zou et al [63], 2013	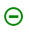	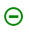	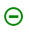	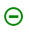	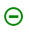		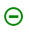	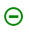	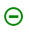		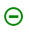
Karamanli et al [64], 2016	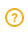	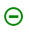	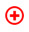	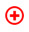	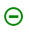		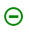	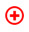	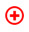		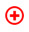
Tawaranurak et al [65], 2021	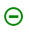	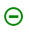	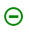	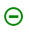	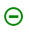		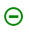	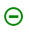	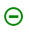		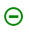
Park et al [66], 2021	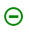	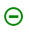	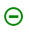	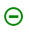	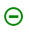		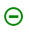	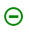	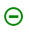		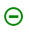

^a^Indicates an unclear risk of bias or concerns regarding applicability.

^b^Indicates a low risk of bias or concerns regarding applicability.

^c^Indicates a high risk of bias or concerns regarding applicability.

### Prediction Models With External Validation

A total of 2 studies [67,68], one in 2000 and another in 2006, performed the external validation of 5 prediction models. The first was a prospective study that evaluated 4 clinical prediction models [12,15,55,57] for predicting the presence of OSA (AHI≥10). They included 370 patients with suspected OSA who underwent PSG between July 1996 and October 1997. The achieved prevalence of OSA was 67%, and the results are shown in Figure 1 and Table 4 of the original article [67]. The highest AUC, sensitivity, and specificity reached were 74%, 96%, and 54%, respectively. The second study used 80 patients with suspected OSA to evaluate the model described in the study by Kushida et al [58]. The objective was to evaluate the clinical applicability and define a clinical cutoff to differentiate OSA severities. Although the authors stated that the clinical applicability exists, they could not define a threshold for clinical use, and they did not present any discrimination measures.

The study of Flemons et al [15], in addition to producing a new prediction model, also applied the 2 equations from studies by Crocker et al [55] and Viner et al [12] to the obtained data set. Although no actual values were presented, the authors stated that the AUCs were very similar.

Furthermore, the study by Flemons et al [15] was externally validated by Khoo et al [62], with 52 patients with suspected OSA, reaching an AUC of 69%. If a clinical threshold of 60% is defined, the model in this independent sample reached 78% sensitivity and 45% specificity.

## Discussion

### Principal Findings

The AASM guidelines [1] explicitly state that *“clinical prediction algorithms may be used in sleep clinic patients with suspected OSA but are not necessary to substitute the need for PSG,”* whereas *“in non-sleep clinic settings, these tools may be more helpful to identify patients who are at increased risk for OSA.”* The evaluation of these tools in a nonsleep clinic setting was not tackled by AASM experts, as it was beyond the guideline scope. Therefore, our work aimed to answer this question by complementing step 1 in the clinical algorithm developed for clinical suspicion of OSA using clinical prediction algorithms in a nonsleep setting. With this, we hope to estimate the probability that OSA is present in a population with suspected OSA that is not yet diagnosed by aggregating information from multivariable prediction models, stating the ones that are best at rule out and rule in.

As such, the studies that only developed a model are the ones that need to gather evidence on whether the model would be helpful to put into clinical practice (high overfitting). To do so, it is needed to validate the model in a new population data set. One way to do this is by splitting the data set or performing a validity assessment using different techniques, such as cross-validation or bootstrapping, or even better, by applying the algorithm to an independent sample.

Of the 63 included studies, only 14 (22%) performed both development and external validation or only external validation of the algorithm. Most selected studies only developed 36% (23/63) or developed and internally validated 41% (26/63) of prediction models.

The study by Zerah-Lancner et al [60] emerged as the best at rule-out OSA, described a sensitivity value of 100% for an OSA definition of AHI≥15. The predictive variables included were respiratory conductance and oxygen saturation, chosen from an external population of 101 participants. The best at rule-in OSA was the study by Pillar et al [56]; for a validation population of 155 participants, it demonstrated a specificity of 94% for an AI≥10 symptoms, with witnessed apneas, NC, age, and falling asleep easily as predictive variables. Both studies used logistic regression as the machine learning approach. The study by Kushida et al [58] reached maximum specificity, but the authors did not describe whether the obtained results were for testing or external validation, in a 300-participant validation data set. These 2 best models [56,60] were developed and validated in 2000 and 1992, respectively, and presented a high risk of bias and applicability, with none of the studies providing the discriminatory power of the model or metric CIs.

The most recent study by Park et al [66], performed in 2021 with a validation data set of 916 participants (largest sample), only reached values of 78% and 76% for sensitivity and specificity, respectively, when compared with the 2 previous best models. This was also a logistic regression, electing BMI, age, sex, Berlin questionnaire score, and tonsil grade as the clinical factors for an OSA definition of AHI≥5. Although this study continued to lack the reporting of study design or prevalence of OSA, it presented a low risk of bias and applicability. But it only included Asian patients, so it cannot be race generalized, as the authors mention.

### Strengths and Limitations

It is important to consider some of the limitations and strengths of our methods and those of the included clinical studies. Although we cannot be sure that we retrieved all published literature, we are confident that our methodology is adequate. Risk was minimized by performing the search in 3 search engines (1 related to health sciences and 2 others with broader spectrums) and in 2 periods.

The PROBAST demonstrated that we face a high risk of bias and applicability, even when only assessing external validation results. Almost all the studies do not report the study design, which can raise problems in generating absolute probabilities or even in terms of inappropriately including or excluding participants. In addition, the definition and measurement of predictors and their association with the outcome were high in the 2 studies, as some of the predictors were not available when the model was intended to be used. Although all outcome definitions were based on PSG, some did not report how the measure was calculated or selected different cutoff values than the ones described in the guidelines. While all studies used appropriate statistical analysis, some lacked a reasonable number of participants with the outcome, in the test or validation data sets. Information regarding exclusion criteria or handling of missing data was not described, and most studies selected predictors based on univariable analysis. Besides all participants who underwent the gold standard exam, some did not have suspected OSA as the only inclusion criterion.

Different approaches have been followed since 1988 with the aim of predicting whether OSA is present in an individual, contributing to unlocking the bottleneck of in-hospital screening or diagnosis. However, assessing the bias or applicability of these approaches is not an easy task, with only 3 studies presenting an overall low risk of bias and applicability [63,65,66]. Furthermore, common missing points need to be pointed out are (1) most studies did not report the study design or period; (2) OSA definition differed within time, guidelines, and studies; (3) OSA prevalence varied from 30% to 93%, with some studies not describing the proportion; (4) needed measures to assess diagnostic value such as sensitivity, specificity, and AUC are not reported, and when reported, did not present CIs; and (5) some studies only create the predictive model and others add the validation task, but external validation is still lacking in all the studies.

Regarding the chosen machine learning approaches, the most common was logistic regression (35/63, 56%), followed by linear regression (16/63, 25%), support vector machine (9/63, 14%), neural networks (8/63, 13%), decision trees (8/63, 13%), Bayesian networks (4/63, 6%), random forest (2/63, 3%), discriminant analysis (2/63, 3%), classification and regression tree (2/63, 3%), nomogram (2/63, 3%), Pearson correlation (1/63, 2%), adaptive neuro-fuzzy inference system (1/63, 2%), artificial immune recognition system (1/63, 2%), genetic algorithm (1/63, 2%), supersparse linear integer models (1/63, 2%), and the k-nearest neighbors algorithm (1/63, 2%).

### Conclusions

In summary, this review provides an extensive, comprehensive, and up-to-date synthesis of diagnostic models in OSA. It is possible to predict OSA by only taking into consideration simple and available predictors such as BMI, age, sex, or NC as well as by reaching high levels of sensitivity or specificity, depending on whether we want to elect a rule-out or rule-in approach.

## Abbreviations

AASM: American Academy of Sleep Medicine
AHI: apnea-hypopnea index
AUC: area under the receiver operating characteristic curve
NC: neck circumference
OSA: obstructive sleep apnea
PROBAST: Prediction Model Risk of Bias Assessment Tool
PROSPERO: International Prospective Register of Systematic Reviews
PSG: polysomnography
